# Effects of 12 weeks of Tai Chi Chuan intervention on the postural stability and self-reported instability in subjects with functional ankle instability: Study protocol for a randomized controlled trial

**DOI:** 10.3389/fneur.2022.923669

**Published:** 2022-09-21

**Authors:** Xiao-hua Ke, Dun-bing Huang, Yin-yan Li, Xiao-mei Li, Jin-hua Guo, Miao-miao Guo, Sheng-xian Yu, Sheng-chao Ma, Cai Jiang, Zhong-hua Lin

**Affiliations:** ^1^Department of Rehabilitation Medicine, Shanghai Fourth People's Hospital Affiliated to Tongji University School of Medicine, Shanghai, China; ^2^Rehabilitation Center, Zhejiang Hospital, Hangzhou, China; ^3^General Outpatient Department, Fujian Academy of Chinese Medical Sciences, Fuzhou, China; ^4^Shengli Clinical Medical College, Fujian Medical University, Fuzhou, China; ^5^The Second Rehabilitation Department, Fujian Provincial Hospital, Fuzhou, China; ^6^Fujian Institute of Clinical Geriatric, Fujian Provincial Hospital, Fuzhou, China

**Keywords:** Tai Chi Chuan, functional ankle instability, postural stability, self-reported instability, protocol

## Abstract

**Background:**

Tai Chi Chuan (TCC) is a physical activity modality that originated in China and is now widely popular around the world. Although there are a series of articles reporting that TCC can improve balance and other functional symptoms in a variety of populations, including the elderly, patients with stroke, and patients with Parkinson's disease, its efficiency has not been scientifically and methodically evaluated in subjects with functional ankle instability (FAI). Moreover, there is no literature directly comparing TCC and conventional balance training (CBT) interventions for FAI. The objective of this study is to investigate the comparative effects of TCC intervention and CBT protocols in improving postural balance and subjective instability feelings in patients with FAI.

**Methods:**

This study will be a single-center, parallel group, randomized controlled trial. Sixty-eight patients with FAI will be included and randomly assigned in a 1:1 ratio to either an intervention group (*n* =34) or a control group (*n* = 34). The participants in the intervention group will complete 12 weeks of TCC intervention (40 min/time, 3 times/week for 12 weeks) on the basis of health education treatment. The control group will receive health education and 36 CBT sessions during a 12-week period. Outcome measures include postural stability and self-reported feelings of instability at baseline, after the end of the intervention, and 3-month follow-up. The postural stability assessment of patients with FAI will be detected by performing static and dynamic postural tests, which will be carried out through a specific balance platform (TecnoBody ProKin). Self-reported feelings of instability will be assessed by Cumberland Ankle Instability Tool (CAIT), American Orthopedics Foot and Ankle Society's Ankle–Hindfoot Evaluation Scale (AOFAS-AHES), and the MOS item Short Form Health Survey (SF-36).

**Discussion:**

This trial will demonstrate whether a 12-week TCC intervention positively affects postural stability and self-reported outcomes in patients with FAI. At the same time, the superiority of its clinical efficacy will also be compared with that of CBT. This study may also help to redefine the value of traditional Chinese exercises in the treatment of chronic ankle instability.

**Clinical trial registration:**

Chinese Clinical Trial Registry: ChiCTR2100041790. Registration date: 22 March 2021. http://www.chictr.org.cn/edit.aspx?pid=119501&htm=4.

## Background

Ankle inversion sprain is one of the most common injuries suffered in both sportsmen and physically active individuals (1). It has been reported that the recurrence rate of a lateral ankle sprain after the initial injury is as high as 80% (2). Recurrent ankle sprains often result in residual symptoms and often lead to the development of functional ankle instability (FAI) (3). FAI is one of the leading causes of people's daily activity disorders, which is characterized by recurrent ankle instability, a feeling of “giving way,” persistent ankle pain, and self-reported disability (4, 5). The pathogenesis of FAI is thought to be associated with mechanical impairment, neuromuscular, and sensorimotor deficiencies, such as decreased proprioception, strength deficits, and motor neuron excitability (6, 7). All these symptoms can lead to postural control impairment and ankle instability in patients with FAI, severely affecting mobility and quality of life in patients with FAI (8–10).

Balance ability refers to the ability of the human body to maintain the stability of the center of gravity in the process of static and dynamic movement, and the decline of balance ability will increase the instability of the ankle and risk of ankle injury (11). Previous studies have shown that balance training, proprioceptive exercise, and manual therapy can be used to improve ankle function and increase ankle stability (12–14). Among these, balance training is a widely used intervention in clinical practice which can improve posture control ability, motor ability, proprioception function, and increase the stability of ankle joints (13).

Tai Chi Chuan (TCC) is a traditional Chinese exercise form known for its slow and graceful movements, which is widely used around the world. TCC emphasizes abdominal breathing, waist axis driving limb movement, virtual and real conversion of center of gravity, and coordinated swing of hip/knee/ankle to improve body stability, as well as forward and backward stepping movements to increase the ranges of dorsiflexion and plantarflexion (15). Studies have shown that the practice of this physical activity can produce a series of beneficial effects, such as improvement of proprioception, activation of core muscle groups, and improvement of muscle strength around joints, all of which may have a positive impact on patients with chronic ankle instability (CAI) (16, 17). Although self-reported improvements in functional status, postural balance, and lower extremity strength have been demonstrated in response to TCC, to the best of our knowledge, there is still a lack of effective evidence that postural control improvements occur as a result of practicing TCC in individuals with FAI.

As far as we know, only one research group has reported that TCC intervention has a positive effect on postural control and self-reported instability feeling in patients with CAI but failed to compare the clinical effects with other training programs (18). Moreover, there is no literature directly examining the comparative effects of TCC intervention and conventional balance training (CBT) protocols regarding their impact on postural stability and self-reported outcomes in patients with FAI. From the perspective of clinical utility, it is extremely interesting to see which intervention is more effective as clinicians always want to be able to select the best intervention for patients with FAI. Theoretically, patients with FAI are a series of complex clinical syndromes resulting from the interaction of pathomechanical, sensory perceptual, and motor behavior impairments (19, 20). Balance training alone may only achieve partial therapeutic effects. TCC is a kind of mind–body exercise that may be particularly beneficial to FAI because it involves movement recall, task switching, attention, and visuospatial processing simultaneous with physical movements (21). Therefore, our hypothesis is that both TCC intervention and CBT would obtain significant improvements, with a possible superiority of TCC over CBT. Thus, the aim of this randomized controlled study is to test the comparative effects of TCC intervention and CBT protocols in improving postural balance and subjective instability feelings in patients with FAI.

## Methods and analysis

### Study design and setting

This study will be a single-center, randomized, exploratory trial with two parallel groups. The objective is to test the comparative effects of TCC intervention and CBT protocols in improving postural balance and subjective instability feelings in patients with FAI.

**Figure 1:**
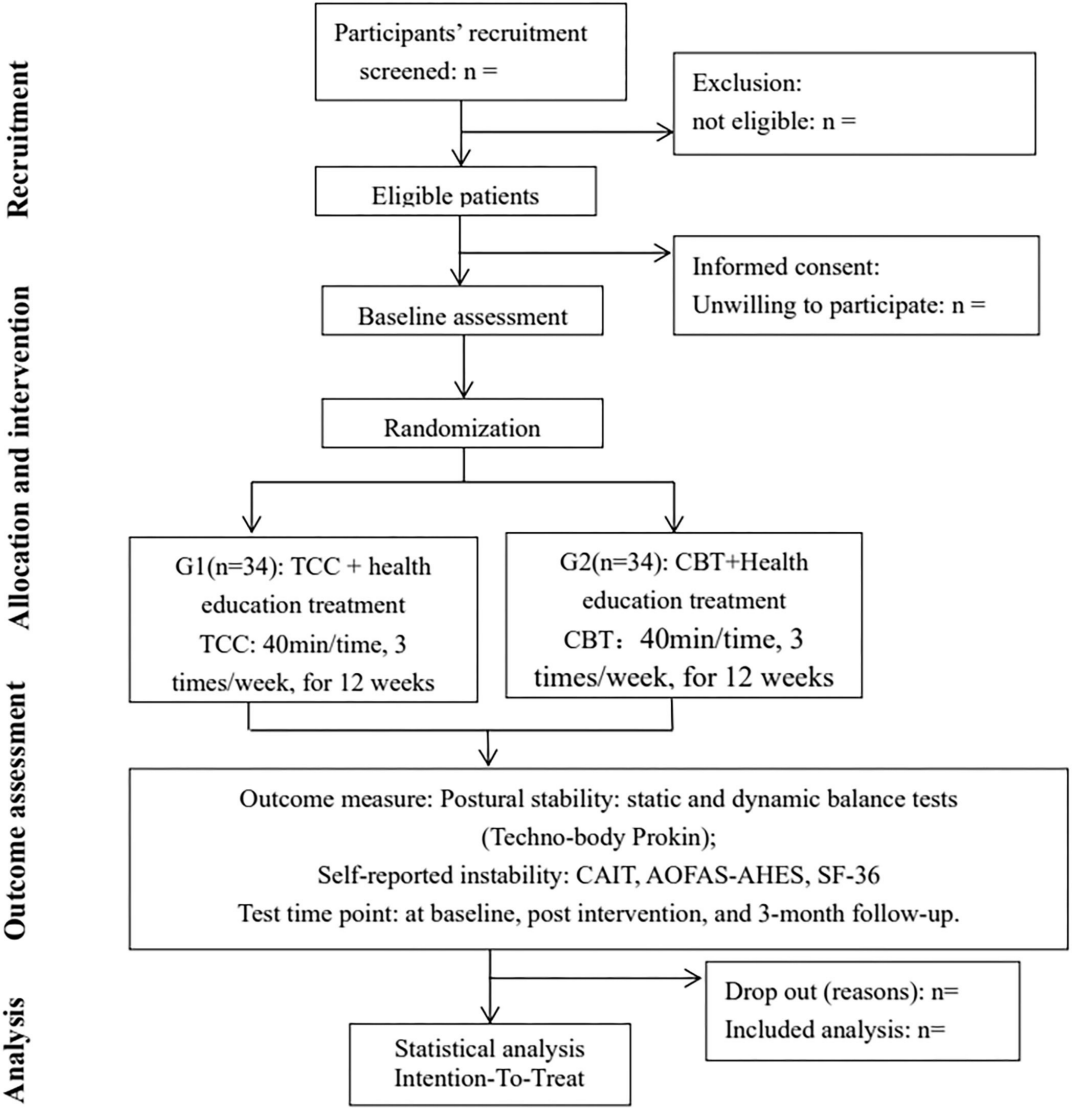
Flow diagram of participants.

We recruited subjects from the Fujian Provincial Hospital, the surrounding Sports Center, and residents of Gulou District, Fuzhou City, Fujian Province, China. Potential eligible subjects who meet the following inclusion criteria but are excluded from the exclusion criteria will be determined by two orthopedic surgeons for eventual inclusion in the study. After signing the written informed consent form, eligible participants will be randomly divided into two groups, the intervention group or the control group, at a 1:1 ratio. All assessments will be conducted at baseline, post intervention, and 3-month follow-up. This study was designed in strict accordance with the Standardized Protocol Items, including the Recommendations for Interventional Trials (SPIRIT) Checklist, and has been approved by the Ethical Committee of Fujian Provincial Hospital (no. k2019-03-035). In addition, we have completed registration in the Chinese Clinical Trials Registry in 2021. The flow diagram for this trial is shown in Figure 1. The specific flowchart of participant recruitment is shown in Table 1.

**Table 1:** Trial processes chart.

**Items**	**Before enrollment−2 to 1 (week)**	**Intervention period 1−12 (week)**	**Outcome assessment 13 (week)**	**3-month follow-up**
Inclusion criteria	√			
Exclusion criteria	√			
Informed consent	√			
Baseline	√			
Randomization and allocation	√			
Intervention		√		
Static balance tests	√		√	√
Dynamic balance tests	√		√	√
CAIT	√		√	√
AOFAS-AHES	√		√	√
SF-36	√		√	√
Adverse events		√		
Reasons of drop-out and withdrawals		√		

### Inclusion criteria

Participants who meet the following criteria will be included: (1) a history of one or more lateral ankle sprains within at least 6 months prior to the participation in this study; (2) a score on the Cumberland Ankle Instability Tool (CAIT) that is ≤ 25 points ensures a sense of ankle instability; (3) no history of other bone, muscle, and joint injuries of lower limbs; (4) no history of other neurological diseases, such as those that alter cognition, ataxia, and balance dysfunction; (5) pain in the ankle below the mild level, visual analog scale (VAS) ≤ 3; (6) those physically active and able to maintain their activity level regardless of their FAI status; and (7) those willing to participate and signed the informed consent form.

### Exclusion criteria

Participants who meet the following criteria will not be included in this study: (1) those with particular diseases in addition to ankle instability; (2) those who have experienced ankle joint surgery in the past; and (3) those who have participated in an ankle rehabilitation program 6 weeks prior to this study.

### Withdrawal from the study

In any of the following cases, participants will be allowed or asked to withdraw from the study:

(1) Those unwilling to continue participation in the experiment for any reason no matter at what stage of the trial.
(2) Those who experience an adverse event (AE) that it is not suitable for continued participation in the trial.
(3) Participants absent from TCC training too many times and not suitable for continued participation in the trial.

### Sample size

Sample size calculations were done using the G-power 3.1.9.2 software (developed by the University of Düsseldorf, Germany). An effect size of 0.8 and a two-sided alpha level of 0.05 were set based on the differences of pre- and post-treatment repeated measurement points of Star Excursion Balance Test (SEBT) and CAIT; a total sample of 56 participants (28 patients in each group) is required to achieve 90% power. The estimated withdrawal rate was 10–20%, and a total of 68 participants (34 patients in each group) were planned to be enrolled in this clinical trial. The power analysis is based on the primary outcome variable, including within-group differences in SEBT and CAIT (dependent variables) before and after treatment. The means and standard deviations of the SEBT and CAIT are based on the results from a similar published study from Spain (18) and preliminary experiment of the research group (intervention group: ▴SEBT-posteromedial M = 6.46, SD = 6.89, ▴CAIT M = 10.8, SD = 2.21, control group: ▴SEBT-posteromedial M =0.64, SD = 3.86, ▴CAIT M =0.2, SD = 2.10), with a 1:1 allocation ratio.

### Randomization and blinding

A total of 68 eligible FAI participants will be randomized 1:1 to an intervention group or a control group by simple randomization. Random sequences will be computer-generated through the SAS Statistics software version 9.4 (SAS Institute Inc. Cary, NC) by an independent researcher, who will not be involved in baseline data collection, clinical interventions, functional outcome assessments, data collection, or statistical analysis. The allocation of participants will be concealed through enclosing assignments in sequentially numbered, opaque-sealed envelopes. Clinicians will determine whether subjects can participate in the study in strict accordance with the above inclusion and exclusion criteria. All subjects who agreed to participate and determined that they were suitable to participate in the study will be randomized. The researchers responsible for recruitment will assign the eligible number and in turn receive a closed opaque envelope with randomization numbers, allocation, and intervention information inside. Then they give the envelope to the clinician or therapist. It should be noted that the researchers responsible for recruiting subjects will not be allowed to know about the group assignments. The clinical intervener will open the envelope and learn about the intervention plan to be administered to the participants. Due to the nature of the clinical intervention, the participants, clinician, and therapist cannot be blinded after assignment to intervention. The outcome assessors and data statisticians will not know the treatment allocation until all data analyses have been completed.

### Interventions

#### Control group

Subjects in the control group will receive CBT based on the Single-Legged Balance Training Program. The program consisted of a warm-up exercise, the single-legged stance, single-legged stance with bosu ball exercises, single-legged stance with kicking against resistance in four directions, and step-down with single-legged in four directions. The training difficulty of this exercise prescription is from easy to difficult. In the training process of this experimental study, the subjects were required to keep the body center of gravity stable during the training process, 40 min each time, 3 times a week for 12 weeks.

In addition to the CBT, all participants will receive health education at the start of the study and monthly throughout the study period. FAI is mainly characterized by recurrent “muscle weakness” of the ankle joint associated with ankle pain, muscle strength weakening, proprioception decline, and random control disorders of joint movement. Therefore, it is necessary to inform them of the preventive measures for ankle sprain, instruct them to properly rest, correct their bad exercise patterns (avoid acute jumping, climbing mountains, and other activities that damage joints), and wear ankle protection when necessary. In addition, patients with ankle joint injury are often anxious and corresponding psychological nursing can be carried out to relieve the anxiety and worry of patients.

### Experimental group

On the basis of health education treatment in the control group, the TCC intervention group will undergo a 40-min 24 Simplified Yang-style TCC exercise. Yang-style TCC consists of slow, smooth, and rhythmic movements that emphasize the practitioner's focus on trunk rotation, weight shifting, coordination, and maintenance of lower limb posture stability. Subjects with FAI will be taught and supervised by an expert TCC coach to ensure the unity of practice time and the basic correctness of action rhythm. The complete set of TCC exercises consists of three parts: 5-min warmup, 30-min TCC practice, and 5-min cool down. Practice will occur once per day, three times a week for 12 weeks, for a total of 36 times.

During the whole training process, patients are encouraged to keep practicing if they have slight ankle pain. If the ankle joint pain of the subject is obvious during the training, i.e., VAS > 3 points, we will suggest the subject to rest or suspend the training, and continue after the pain is relieved.

### Demographic parameters

Before all subjects are enrolled, we will collect their demographic characteristics, including age (year), gender (male, female), height (cm), weight (kg), body mass index (kg/m^2^), marital status (single, married, divorced), occupational status (full-time worker, part-time worker, unemployed), affected ankle (left, right), Baseline Cumberland Ankle Instability Tool score, no. of sprains, initial ankle sprain evaluated by a medical professional (yes, no), time since first sprain (year), and time since last sprain (month).

### Outcome measurements

Outcome measures will include postural stability and self-reported instability feelings that will be performed at baseline, post-intervention, and 3-month follow-up.

## Patient-reported outcomes

### Cumberland ankle instability tool

The Cumberland Ankle Instability Tool (CAIT) (22) is used to focus on the severity of functional problems in patients with ankle instability, which is recommended by the International Ankle Consortium. It consists of nine items and is scored on a 30-point scale. Previous research shows that the Chinese version of CAIT is a valid and reliable questionnaire tool to evaluate ankle function in clinical trials. CAIT scale can distinguish between patients with and without CAI. A higher response score represents a better level of overall function and ankle stability, and values ≤ 25 represent ankle instability. According to a recent study, the minimum detectable change score of CAIT is ≥3, which can be used to determine the effectiveness of CAI interventions. Thus, in this study comparisons of CAIT scores before and after treatment are used as the primary outcome measure of functional improvement in patients with FAI.

### American orthopedics foot and ankle society's ankle–hindfoot evaluation scale

American orthopedics foot and ankle society's ankle–hindfoot evaluation scale (AOFAS-AHES) (23) is specifically used to estimate clinical problems of the ankle–hindfoot. The cultural adaptation and validation of the Chinese version of AOFAS-AHES has also been successfully implemented. It includes subjective or patient-reported items, as well as objective or physician-assessed items, on a scale of 0–100, with higher scores indicating better function. According to the scoring results, it can be divided into four grades: excellent (90–100 points); good (75–89 points); acceptable (50–74 points); and poor (< 50 points).

### The MOS item short form health survey

Short form health survey (SF-36) (24) is a concise health questionnaire developed by the Boston Institute of Health in the United States, and the Chinese version of SF-36 has been widely used in clinics. It is widely used to measure the quality of life of the general population as well as in the evaluation of the effect of clinical trials and the evaluation of health policies. The SF-36 is a concise health questionnaire that comprehensively summarizes the respondents' quality of life from eight dimensions: physiological function, physical pain, general health status, energy, social function, emotional function, and mental health. It has been reported that SF-36 is a general health assessment tool that can assess the impact of ankle-related diseases on their quality of life.

### Static balance

In this study, TecnoBody ProKin (ProKin Software Stability, TecnoBody S.r.l., Dalmine, 24044 Bergamo, Italy) will be used to evaluate the static postural control ability of the affected side's foot in patients with FAI. Subjects will be asked to stand on a balanced platform and pressure sways in all directions will be detected. First, subjects will be instructed to look directly at a front screen surface, and then they will be asked to try to maintain their balance on both the legs for 60 s, initially with their eyes open and then closed. Subsequently, patients will be asked to try to maintain their balance for 60 s on the affected side leg for 60 s with their eyes open and then closed. We will obtain the change of center of pressure (COP) of the lower limbs from the subjects. Among these, the static balance ability indexes include the length (mm) and average speed (mm/s) of body sway [total and along the anteroposterior (AP) and mediolateral (ML) axis] and area of body sway (area of the ellipse; mm^2^).

The total length of body swing (perimeter; mm) was calculated as the total length of the distance the center of gravity movement route recorded by the system during the subject's body swing, reflecting the total distance and shaking degree of the center of gravity of the human body. The greater the value, the worse the stability. Area of body sway (area of the ellipse; mm^2^) refers to the area of the center of gravity movement of the human body. The larger the area, the worse the stability.

### Dynamic balance

This study will use a posturographic balance platform to perform dynamic balance tests to evaluate proprioception. The ProKin used in this study is an advanced technology that combines classic tilting platform connected to monitors and speakers. By creating visual and audio feedback, it can respond to the minimum movement of the platform on all planes (25). The movable balance platform in this system can detect balance measurement in all directions with an operating angle of 15°C. The index of dynamic balance ability is the total offset index, which refers to the angle between the position of the patient's overall offset and the vertical midline. The average trace error (ATE) was calculated for the proprioception index. ATE = (track length traced by the patient's ankle foot control cursor – ideal track length)/ideal track length. The greater its value, the worse the posture control ability. Also, 0–35% of ATE is considered very good, 35–100% is considered sufficient, and >100% is considered to indicate problems in proprioceptive control (26).

### Assessment of safety

TCC is a traditional sports event in China. As it is a non-invasive intervention method and the TCC in this study is conducted under the guidance of an expert coach, its standardization and safety are sufficiently guaranteed. Nevertheless, during the study period, if the subject has any discomfort, or new changes to their condition, or any unexpected circumstances, whether related to the study or not, relevant information will be recorded in detail in the case report forms (CRFs). At the same time, researchers will identify the relevance of AEs to clinical interventions. In case of adverse reactions, the clinician will comprehensively evaluate the subject's condition and then decide whether to stop the trial. If any serious adverse event (SAE) occurs, emergency medical assistance will be sought.

### Data management and monitoring

Before the start of the trial, we will invite all researchers related to the project to participate in work training. This training mainly introduces the whole research process, including explaining the trial protocol, personnel cooperation, standard operating procedures (SOPs) of research operation, and contents of CRF and filling specifications. All experimental data will be carefully double entered into the electronic CRFs by researchers for later data statistics and analysis. To guarantee the accuracy and standardization of data entry, an independent research assistant (RA) will be responsible for managing the quality control of data collection during the trial. We will delete the identity numbers and privacy information of all participants and identify their identities using unique numerical codes to protect the privacy of all subjects. In addition, throughout the study period, the independent Data and Safety Monitoring Board (DSMB) of the Scientific Research Department of Fujian Provincial Hospital will monitor the progress of the trial, safety of the study, and integrity and authenticity of the trial. It is recommended that supervisors also should randomly check CRFs. Any incomplete data will be recorded as unknown, missing, or inapplicable. During the whole intervention process, we will record the withdrawals from the trial in detail, and the data will be recorded in CRF for later statistical analysis.

### Data analysis

Statistical analysis will be carried out using SPSS Statistics, version 22.0 (SPSS Inc., Chicago, IL, USA). All assigned subjects with available data will be analyzed according to the intention-to-treat principle. Continuous variables, such as CAIT score, age, and disease duration, will be expressed as mean ± standard deviation (SD) or medians (25th to 75th centiles), depending on whether the distribution of variables is normal or skewed. For other categorical data, we will use Mann–Whitney test, chi-square test, or Fisher's exact test for statistical analysis as appropriate. Multiple-measure variables will be analyzed by repeated-measures ANOVA. Additionally, we will use the last-observation-carried-forward method to deal with the missing data. Statistical significance will be assessed as *p* < 0.05.

## Discussion

Ankle sprain has a high recurrence rate, and repeated sprain often progress into CAI. TCC is an ancient form of physical activity that originated in China and has become widely popular around the world. Even though the benefits of practicing TCC have been proven in different populations, to date there are not enough qualified studies to confirm the clinical effect of TCC exercise on balance ability and ankle instability in subjects with CAI. This study aims to determine the effect of 12 weeks of TCC intervention on postural stability and self-reported outcomes in patients with FAI. At the same time, the superiority of its clinical efficacy will also be compared with that of CBT.

Considering that CAI may limit the sensorimotor system, which may further limit the ability to maintain posture control (27). Commonly used rehabilitation balance programs are reported to improve “proprioception,” which means that they may have a positive effect on the somatosensory system (28, 29). Moreover, as mentioned above, CBT can improve ankle function and increase ankle stability, which has been confirmed by a number of studies (1, 13, 30).

In TCC practice, the slow, gentle, rhythmic movements are connected together in a continuous sequence, and movement transitions, center of gravity shifts, and posture transitions are all closely linked and coherent. These movements challenge the balance control system, which requires the human body to be aware of the body's position and maintain the center of gravity within a changing base of support. Previous studies have indicated that the practice of TCC can effectively improve balance because it can reinforce the sensorimotor system, lower extremity muscle strength, endurance, proprioception, and body awareness (31, 32). In view of the fact that a series of articles have reported that TCC can improve the balance function and other functional symptoms of a variety of populations, including core stability, posture control ability, and lower limb movement ability such as hip, knee, and ankle (16, 32). Thus, we hypothesized that both TCC intervention and CBT would obtain significant improvements of postural stability and self-reported outcomes in patients with FAI.

In addition, several studies have shown that decreased cognitive function may be associated with CAI and decreased postural control may be also related to altered cognitive processing (33, 34). Researchers (35) found that patients with CAI had relatively lower cognitive functions such as composite memory, visual memory, working memory, and attention compared to controls. Another study based on computerized neurocognitive testing showed that basketball players with CAI had slower reaction time, suggesting that CAI may be related to cognitive processing speed and accuracy (36).

TCC is a medium-intensity form of physical and mental exercise that involves cognitive activities such as movement recall, task switching, concentration, and visuospatial processing that are carried out simultaneously with physical movements. Previous studies have reported that TCC training can improve brain executive function, language function, learning and memory ability, and cognitive processing speed (37–39). As TCC is a combination of cognitive and motor tasks, we firmly believe in our hypothesis that 12-week TCC intervention has a positive effect on postural stability and self-reported outcomes in patients with FAI, with a possible superiority over CBT.

## Trial status

Protocol number: version 2.0, 20 January 2021. Recruiting start date: 5 November 2022. Expected study completion date: December 2025. In case of any changes to the protocol, we will notify the relevant parties (investigators, participants, etc.) through email or letter.

## Ethics statement

The studies involving human participants were reviewed and approved by the Ethics Board of Fujian Provincial Hospital (K2019-03-035). The patients/participants provided their written informed consent to participate in this study.

## Author contributions

Z-hL, CJ, X-hK, and D-bH conceived of the study, designed the study protocol, and drafted the manuscript. X-hK, D-bH, and Y-yL wrote the manuscript. Z-hL and CJ are in charge of coordination and direct implementation. J-hG, M-mG, X-mL, S-cM, and S-xY helped to develop the study measures and analyses. All authors contributed to drafting the manuscript, read, and approved the final manuscript.

## Funding

This study was sponsored by the Joint Funds for the Innovation of Science and Technology, Fujian Province (Grant number: 2019Y9026); National Natural Science Foundation of China (Grant number: 81904271); and Fujian Provincial Health Technology project (Grant number: 2019-ZQNB-2).

## Conflict of interest

The authors declare that the research was conducted in the absence of any commercial or financial relationships that could be construed as a potential conflict of interest.

## Publisher's note

All claims expressed in this article are solely those of the authors and do not necessarily represent those of their affiliated organizations, or those of the publisher, the editors and the reviewers. Any product that may be evaluated in this article, or claim that may be made by its manufacturer, is not guaranteed or endorsed by the publisher.

## Abbreviations

FAI: functional ankle instability
CAI: chronic ankle instability
TCC: Tai Chi Chuan
CBT: conventional balance training
CAIT: Cumberland Ankle Instability Tool
SEBT: Star Excursion Balance Test
AOFAS-AHES: American Orthopaedics Foot and Ankle Society's Ankle-Hindfoot Evaluation Scale
SPIRIT: Standardized Protocol Items, Recommendations for Interventional Trials
SF-36: the MOS item Short Form Health Survey
AEs: adverse events
SAE: serious adverse event
COP: center of pressure
ATE: average trace error
RA: research assistant
DSMB: Data and Safety Monitoring Board
CRFs: case report forms
SOPs: standard operating procedures
CONSORT: Consolidated Standards of Reporting Trials
SD: standard deviation
GEE: generalized estimation equations.
